# Bilateral synchronous percutaneous nephrolithotomy in single session: A case from a district hospital

**DOI:** 10.1016/j.eucr.2025.103152

**Published:** 2025-08-06

**Authors:** Mohamad Rheza Firmansyah, Andhika Hernawan Novianda, Widi Atmoko, Octoveryal Aslim

**Affiliations:** a Department of Urology, Cipto Mangunkusumo General Hospital, Universitas Indonesia, Jakarta, Indonesia; b Department of Urology, Ibu Fatmawati Soekarno District Hospital, Surakarta, Indonesia

**Keywords:** Percutaneous nephrolithotomy, Single session PCNL, Kidney calculi, Bilateral renal stones

## Abstract

Kidney stones are the most common urological condition worldwide, with rising prevalence. Bilateral cases present challenges such as longer surgery, anesthesia risks, and extended hospitalization. We report a case of a 59-year-old female with bilateral hydronephrosis and multiple stones, successfully treated with bilateral synchronous percutaneous nephrolithotomy (PCNL) at a district-level hospital in Surakarta, Indonesia. Using existing nephrostomy access and meticulous planning, the procedure minimized operative time and blood loss. Full stone clearance was achieved without complications, and renal function remained stable. Bilateral synchronous PCNL is a cost-effective, efficient option in selected patients, requiring advanced surgical expertise and well-coordinated care.

## 1 Introduction

Kidney stones represent the most prevalent condition in urology worldwide. The prevalence of kidney stones increased steadily from 6.5 % in the 2007–2008 cycle to 9.4 % in the 2017–2018 cycle. The prevalence was 10.9 % in men compared to 9.5 % in women.^1^

The prevalence of kidney stones in West Asia, Southeast Asia, South Asia, South Korea, and Japan is 5–19.1 %. These areas are considered to form a ‘stone belt.^2^

In the past, treating bilateral kidney stones came with drawbacks such as long surgery times, the necessity for additional anesthesia, increased risk of bleeding, and extended hospital stays. As a result, significant efforts have been directed towards developing methods to address stones on both sides in a single procedure.^3^

The goal of this paper was to talk about our experience with bilateral synchronous percutaneous nephrolithotomy (PCNL) at district hospital at Surakarta, Indonesia.

## 2 Case presentation

**Fig. 1:**
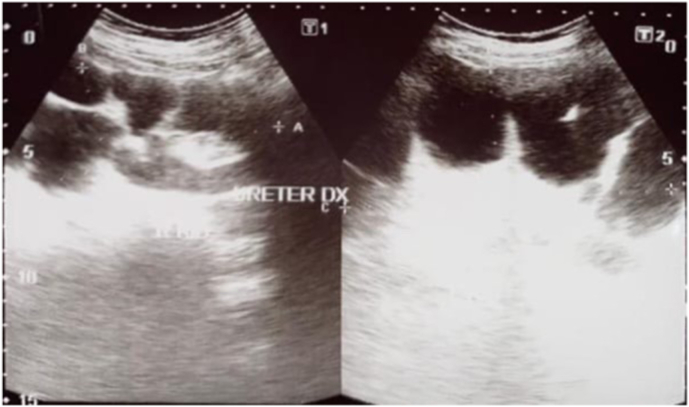
Ultrasonography examination showing hydronephrosis on both kidney.

A 59-year-old female was referred to our centre because of bilateral hydronephrosis from ultrasonography (Fig. 1). For approximately two months, the patient has been experiencing ongoing pain in the flank areas on both sides, with the condition deteriorating over the past week. The blood analysis shown increase in ureum, creatinine and leucocytes level (Ureum 142mg/dL, Creatinine 6,6mg/dL, leucocytes 12.800/μL). Bilateral nephrostomy was done to release the obstructive uropathy.

**Fig. 2:**
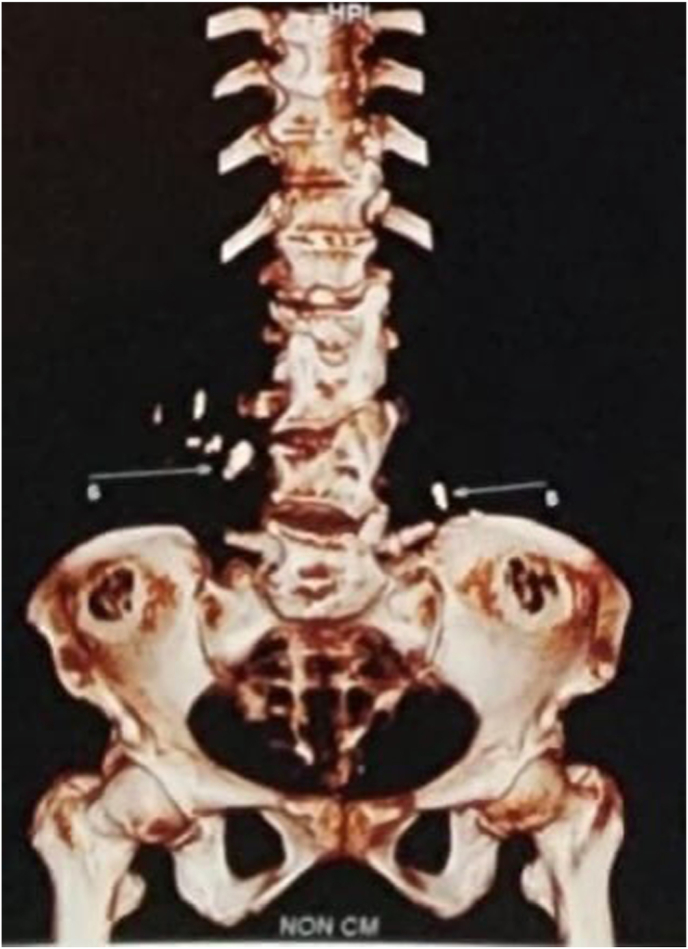
CT scan reconstruction confirming multiple kidney stones and bilateral ureteral stones.

The patient underwent a non-contrast computed tomography (CT) urography, which revealed multiple bilateral renal calculi and proximal ureteral stones (Fig. 2). As the left ureteral stones were located in the proximal ureter, we planned to perform a pushback maneuver followed by synchronous bilateral percutaneous nephrolithotomy (PCNL). Given the patient's remote residence, a single-session procedure was preferred to minimize the need for repeat hospitalization.

**Fig. 3:**
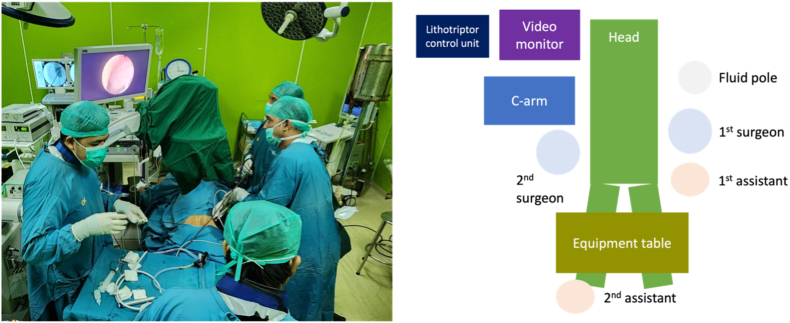
Operating room setup.

Pre-procedure planning included the patient's position, access tract, and the position of the equipment (Fig. 3). After placing bilateral ureteral catheters while the patient was in a supine position, the patient's position was changed to prone. Antegrade pyelography was performed from the nephrostomy to overview the kidney. Positioning the C-arm, monitor, lithotripter, irrigation sets, and other equipment on the left side enables both operators to perform the procedures effectively. Access was established through the existing nephrostomy tract, as antegrade pyelography performed prior to the procedure demonstrated that this route allowed access to all stone burdens. This approach minimized operative time by avoiding the need for an additional puncture. Dilatation was carried out simultaneously, eliminating the need to reposition the C-arm.

A single surgical setup was utilized for the procedure. Stone fragmentation was performed using a pneumatic lithotriptor. We began with the right-sided PCNL, followed by the left side, using the same surgical arrangement. No significant intraoperative bleeding was observed, and no blood transfusion was required. Complete stone clearance was successfully achieved. The patient's postoperative period proceeded smoothly. A KUB radiograph was obtained, as the stones were confirmed to be radio-opaque intraoperatively, making KUB an adequate and cost-effective follow-up tool in this context.

## 3 Discussion

Treating complex renal calculi presents a significant challenge for urologists due to the stones' large size and the intricate techniques required for removal. Percutaneous nephrolithotomy (PCNL) is the preferred method for addressing these substantial and complicated stones, offering a targeted and effective treatment option.^4^

The synchronized method offers multiple benefits. Utilizing dual teams reduces the operation time needed to clear both kidneys, eliminating the necessity for several staged operations. This approach is particularly beneficial for older patients with significant health issues.^5^

For individuals with large stones in both kidneys, bilateral synchronous PCNL could be an option, given certain conditions are met. This approach generally reports very high rates of complete stone removal (95 %–97 %), low rates of complications (9 %–12 %), brief hospital stays (4–6 days), and minimal need for blood transfusions.^6^

For this particular patient, we performed synchronous bilateral PCNL and achieved complete stone clearance. However, according to a recent meta-analysis by Zhu et al., patients who underwent unilateral PCNL demonstrated significantly higher stone-free rates compared to those who underwent bilateral PCNL. The analysis reported an odds ratio of 1.37 (95 % CI: 1.10–1.70, p = 0.004), indicating a statistically significant advantage in favor of unilateral procedures.^7^

No complications were observed during or after the procedure. This is consistent with findings by J. Svihra Jr. et al., who reported that the complication rates for bilateral PCNL are comparable to those of staged unilateral procedures.^8^

We meticulously planned the operating room (OR) layout in advance, drawing on our extensive experience with both our OR setup and the coordination of a highly skilled surgical team. This thorough preparation ensured a smooth and uninterrupted procedural flow, eliminating the need for intraoperative equipment adjustments or repositioning. Our proactive approach to OR organization directly contributed to the efficiency and success of the surgery by anticipating and mitigating potential disruptions before they could occur.

This strategy aligns with the findings of Carrión et al., who emphasized the feasibility and advantages of performing simultaneous bilateral percutaneous nephrolithotomy (SBPCNL) in the prone position without the need for repositioning. Their study, “Prone percutaneous nephrolithotomy: its advantages and our technique for puncture”, highlights that prone SBPCNL is not only safe and effective but also offers several benefits, including single anesthesia exposure, reduced operative time, shorter hospital stay, and decreased psychological stress for patients. These factors, in combination with optimized OR planning, contribute to enhanced surgical efficiency and favorable outcomes in the management of complex bilateral renal calculi.^9^

Postoperative care was provided over a period of three days. While bilateral PCNL may be associated with a modestly longer hospital stay compared to unilateral procedures, multiple studies—including Svihra Jr. et al. and Zhu et al.—suggest this difference is not clinically significant in well-selected patients. Furthermore, bilateral same-session PCNL may reduce total cumulative hospitalization when compared to staged unilateral interventions.^7^^,^^8^

In our experience with bilateral synchronous PCNL, the duration of surgery and anesthesia does not significantly differ from that of unilateral procedures. Although our experience is somewhat limited, the procedure has been shown to be practical. However, its feasibility is constrained by factors such as the availability of necessary equipment and accessories, as well as the expertise of the surgical teams involved.

## 4 Conclusion

Undergoing bilateral synchronous PCNL can notably decrease the overall cost of therapy, hospital stay, need for blood transfusions, risk of infection, and total operation time since only one surgical procedure is required. Despite its considerable advantages, performing bilateral synchronous PCNL is challenging and highly dependent on the surgeon's expertise. It is typically recommended for a select group of patients with straightforward bilateral stones.

## CRediT authorship contribution statement

**Mohamad Rheza Firmansyah:** Writing – review & editing, Writing – original draft, Visualization, Software, Resources, Project administration, Methodology, Investigation, Formal analysis, Data curation. **Andhika Hernawan Novianda:** Validation, Supervision, Formal analysis. **Widi Atmoko:** Validation, Supervision. **Octoveryal Aslim:** Validation, Supervision, Conceptualization.

## Conflict of interest

There is no conflict of interest.
